# Is agriculture driving the diversification of the *Bemisia tabaci* species complex (Hemiptera: Sternorrhyncha: Aleyrodidae)?: Dating, diversification and biogeographic evidence revealed

**DOI:** 10.1186/1471-2148-13-228

**Published:** 2013-10-18

**Authors:** Laura M Boykin, Charles D Bell, Gregory Evans, Ian Small, Paul J De Barro

**Affiliations:** 1 ARC Centre of Excellence in Plant Energy Biology, M315, The University of Western Australia, 35 Stirling Highway, Crawley, Western Australia 6009, Australia; 2 Department of Biological Sciences, University of New Orleans, 2000 Lakeshore Drive, New Orleans, LA 70148, USA; 3 USDA/APHIS/NIS, 10300 Baltimore Ave, BARC-West, Bldg. 005, Room 09A, Beltsville, MA 20705, USA; 4 CSIRO Ecosystem Sciences, GPO Box 2583, Brisbane QLD 4001, Australia

**Keywords:** Whitefly, Aleyrodidae, Molecular clock dating, BEAST, Insect evolution, Fossils

## Abstract

**Background:**

Humans and insect herbivores are competing for the same food crops and have been for thousands of years. Despite considerable advances in crop pest management, losses due to insects remain considerable. The global homogenisation of agriculture has supported the range expansion of numerous insect pests and has been driven in part by human-assisted dispersal supported through rapid global trade and low-cost air passenger transport. One of these pests, is the whitefly, *Bemisia tabaci*, a cryptic species complex that contains some of the world’s most damaging pests of agriculture. The complex shows considerable genetic diversity and strong phylogeographic relationships. One consequence of the considerable impact that members of the *B. tabaci* complex have on agriculture, is the view that human activity, particularly in relation to agricultural practices, such as use of insecticides, has driven the diversification found within the species complex. This has been particularly so in the case of two members of the complex, Middle East-Asia Minor 1 (MEAM1) and Mediterranean (MED), which have become globally distributed invasive species. An alternative hypothesis is that diversification is due to paleogeographic and paleoclimatological changes.

**Results:**

The idea that human activity is driving speciation within the *B. tabaci* complex has never been tested, but the increased interest in fossil whiteflies and the growth in molecular data have enabled us to apply a relaxed molecular clock and so estimate divergence dates for the major lineages within the *B. tabaci* species complex. The divergence estimates do not support the view that human activity has been a major driver of diversification.

**Conclusions:**

Our analysis suggests that the major lineages within the complex arose approximately 60–30 mya and the highly invasive MED and MEAM1 split from the rest of the species complex around 12 mya well before the evolution of *Homo sapiens* and agriculture. Furthermore, the divergence dates coincide with a period of global diversification that occurred broadly across the plant and animal kingdoms and was most likely associated with major climatic and tectonic events.

## Background

Studying agriculture in the context of evolutionary biology is an emerging area of research [1-3] driven in part by the need to more fully understand the biotic interactions between pests and pathogens, and their plant hosts and natural enemies [1]. The need to feed the world through increased productivity and the reduction in pre-harvest losses is driving the need to better understand the interactions between humans, plants and insects. Evolutionary theory is emerging as an important lens through which to explore the adaptive tensions that exist. The majority of studies have, so far, focused on the role human selection has played directly on pests (e.g. the evolution of resistance to insecticides), [4]. However, little attention has been given to the role that human mediated selection in agricultural systems might play on speciation of pests and pathogens. To begin to explore this we combine a relaxed molecular clock and a globally distributed insect species complex that contains some the world’s most damaging pest species that have been spread from country to country by trade in ornamental plants, to explore whether human activity maybe exerting an influence on the level diversification that has been observed within this complex.

Members of the *Bemisia tabaci* species complex are small, sap sucking insects known as whiteflies that are capable of causing extensive damage to major food staples such as cassava and sweetpotato, vegetable, grain legume and fiber crops either through direct feeding or the transmission of plant pathogenic viruses. Our ability to comprehend the levels of diversity within the *B. tabaci* complex has been hampered in part by the lack of any morphological means of separating different members [5,6]. However, during the last two decades, molecular markers have become available that enable us to clearly delimit the different members of the complex. Global relationships have now been addressed in recent phylogenies based on the mitochondrial cytochrome oxidase one gene (mtCOI) [7-10]. These studies have revealed at least 34 distinct genetic groups with sufficient evolutionary distance to suggest that they are separate species [7,8,10-12]. Two of these species, known currently as *Mediterranean* (MED) and *Middle East-Asia Minor 1* (MEAM1), until formal revised description of the complex occurs, are considerable pests of agriculture and have been spread globally through trade in ornamental species [13,14]. Furthermore, the species-level boundaries identified with mtCOI are supported by all available mating studies which show that the different members are mostly unable to copulate and where copulation occurs between the more closely related members of the complex, the resulting fitness of F1 and F2 progeny is considerably inferior to that of the parents [15,16]. The current phylogeny for the *B. tabaci* species complex is now well-resolved and so suitable for analysis using molecular dating.

Molecular dating is a powerful technique for obtaining diversification dates on a phylogeny derived from molecular data. The modern molecular clock is now 48 years old [17] and has been used to estimate divergence dates for many organisms including insects [18-21]. Early criticisms of the molecular clock were directed at the assumption that the rates of evolution were assumed equal across the entire phylogeny [22,23]. However, recent advances have incorporated the ability to specify different rates of evolution across lineages by utilizing a relaxed molecular clock [24,25] thereby making the methods robust to violations of the strict molecular clock. Calibrating molecular clocks is most often done using fossil records, which are used to provide evidence as to when a species first appeared, and when different lineages diverged. Calibrating a phylogeny with the relaxed molecular clock and fossil information has made substantial progress in the last few years with developments in assessing calibration uncertainty [24,26].

*Bemisia tabaci* belongs to the family Aleyrodidae which is comprised of four subfamilies, the Udamosellinae which contains two species, the Bernaeiinae which is made up of four extinct species belonging to three genera, the Aleurodicinae which has 136 species in 21 genera and the Aleyrodinae which has 1492 species in 140 genera (including *Bemisia*). Importantly, there is also a considerable fossil record [27-29], which includes *Aleurodicus burmiticus*. This species was found in Burmese amber from the Lower Cretaceous (125–135 mya) [30] and is the most appropriate fossil calibration point for our Aleyrodidae phylogeny as it is morphologically similar to the basal members of the phylogeny, *Aleurodicus dispersus* and *Aleurodicus dugesii.* Another key fossil is *Baetylus kahramanus* which was recently described from Lebanese amber [27]. *Baetylus kahramanus*[27] is morphologically similar to *Bemisia tabaci* as it has a single, radial vein in the forewing and the male has four wax plates on the venter of the abdomen. Also, the panonychium (on the tarsal claw) of *B. kahramanus* is more like that of the Aleyrodines (*B. kahramanus* has a bilobate panonychium, whereas that of the Aleyrodines is somewhat spatulate with many hairs, and in Aleurodicines it is just a simple spine-like seta).

The origin and radiation of whiteflies was first considered in the early 1990s [31,32], but has received no subsequent attention. Campbell [32] proposed radiation of ancient lineages of the Aleyrodidae occurred in association with the appearance of the angiosperms during the lower Cretaceous and paleogeographic and paleoclimatological changes associated with tectonic activity. The well defined molecular phylogenies for *B. tabaci*[7,11] and fossil data [27] now enables us to test whether hypotheses generated by Campbell are supported. We do this by updating the *B. tabaci* species complex phylogeny with newly available mtCOI sequence data from GenBank and then apply the best practice molecular clock methodology of [33] to explore when diversification within the complex most likely occurred. Through this we will be able to determine whether there is any correlation between human activity and speciation within the *B. tabaci* species complex and more particularly, in the evolution of the two highly invasive members of the complex.

## Methods

### Data collection

The data set consisted of the 657 bp fragment of the 3’ end of mtCOI. The alignment of mtCOI data from Boykin et al. (2012) was used as the starting point for the sequences included in this study; all aligned mtCOI data used in this analysis can be found at the CSIRO data portal (http://dx.doi.org/10.4225/08/50EB54B6F1042) and are publically available. The taxonomy browser in GenBank was used to find outgroups that had sequences available for mtCOI. Outgroup taxa were selected based on their morphological similarities to the fossil *Aleurodicus burmiticus*[27]. In addition to *B. tabaci*, 40 additional outgroup haplotytpes representing 19 species were included in the alignment. All of the mtCOI sequences were aligned in Geneious [34] with the MUSCLE [35] alignment option set to 50 iterations, then visually inspected and manually adjusted where necessary. The final alignment was translated to ensure sequences were aligned within the correct reading frame. All duplicate haplotypes were removed, as were any haplotypes with gaps or ambiguous sequences that exceeded 1% of the sites leaving a total of 537.

### Assessing rate heterogeneity

SymTest was used to test whether the included sequences evolved under the same conditions [36,37]. To further test the rates of evolution in the different species of the *B. tabaci* species complex, Shannon entropy [38] was calculated at http://www.hiv.lanl.gov/. Shannon entropy is a measure of variation in DNA and protein sequence alignments. Entropy has been used to test whether new virus strains (HIV and Hepatitis C) are more variable than the background (older strains), a more variable strain (higher entropy) is indicative of a “break-out” strain [39]. Entropy was calculated for the invasive species MED and MEAM1 because they are the “break-out” species in the complex.

### Phylogenetic analyses

The model of molecular evolution was determined using Modeltest 3.6 [40]. MrBayes 3.1.2 [41] was run in parallel (8 processors) on the BeSTGrid supercomputer in New Zealand [42]. The data were partitioned based on codon position in the alignment. Codon positions 1 and 2 were treated with the following commands in MrBayes: lset applyto = (1,2) nst = 2 rates = gamma and the third position: lset applyto = (3) nst = 6 rates = gamma. MrBayes 3.1.2 was run for 50 million generations and trees were sampled every 1000 generations. All runs reached a plateau in likelihood score (i.e. stationarity), which was indicated by the standard deviation of split frequencies (0.0015), and the potential scale reduction factor (PSRF) was close to one, indicating the MCMC chains converged. Convergence of the runs was also checked using Tracer v1.5.4 [43] and the effective sample size (ESS) values were well above 200 for each run. Twelve thousand five hundred trees were suboptimal at the beginning of the runs and were therefore discarded.

### Divergence estimates

BEAUti v1.7.1 [44] was used to generate the xml file for the multiple BEAST runs. Four independent runs of BEAST were conducted, each consisting of two chains resulting in 8 independent runs. For each run, one taxon set for *Aleurodicus* was defined and forced to be monophyletic, the site model was specified as HKY, base frequencies were estimated, Gamma plus invariant sites was the site heterogeneity model, 4 gamma categories, partitioned into codon positions (1 + 2), 3. The clock model was set to lognormal relaxed clock and the tree prior was set to “Speciation: Birth-Death Process”. The tree height prior was set to a normal distribution, with an initial value of 123, to represent the age of the fossil [27] with a standard deviation of 20. The MCMC were run for 50 million generations and sampled every 1000th generation. The xml files created in BEAUti was then used as input into BEASTv1.7.1 implemented on the BeSTGrid supercomputer. Convergence of the multiple runs was checked using Tracer v1.7.1 [43] and the ESS values were well above 200 for each run. Two independent BEAST runs were completed and the two tre files were combined using Logcombiner [45]. The trimmed output was combined to yield posterior estimates of mean.rate, coefficient variation in rates, ucld.mena, ucld.stdev and the mean and 95% highest posterior density (HPD). TreeAnnotator [46] was used to generate a final tree which was viewed in FigTree v1.3.1 [47].

## Results

### Assessing rate heterogeneity

**Figure 1:**
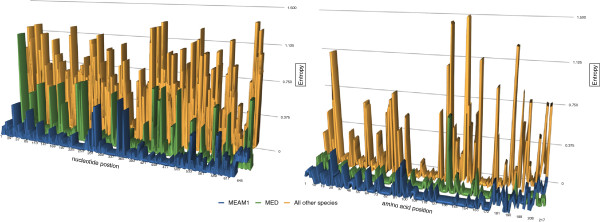
**Shannon entropy**[38]**was calculated at**http://www.hiv.lanl.gov/**.** Shannon entropy is a measure of variation in DNA and protein sequence alignments. Entropy has been used to test whether new virus strains (HIV and Hepatitis C) are more variable than the background (older strains), a more variable strain (higher entropy) is indicative of a “break-out” strain [39]. Entropy was calculated for the invasive species MED and MEAM1 because they are the “break-out” species in the complex.

If diverging sequences have evolved under the same conditions, then uniformly distributed p-values are expected; in other words, we would expect 5% of the tests to produce p-values < 0.05, 1% of the tests to produce p-values < 0.01, and so forth. A slight deviation from the null expectation was observed in our data (data not shown). Figure 1 shows entropy scores for the MED, MEAM1 and all other species at both the NA and AA level. The entropy scores range from 0, which indicates that position in the alignment is identical for all species, to 1.5 indicating there is considerable variation in the NA or AA at that position in the alignment. It is clear from Figure 1 that the other species all have higher entropy scores indicating that the MED and MEAM1 species do not show more variability at both the nucleotide and amino acid levels when compared to the other species in the complex.

### Phylogenetic relationships

**Figure 2:**
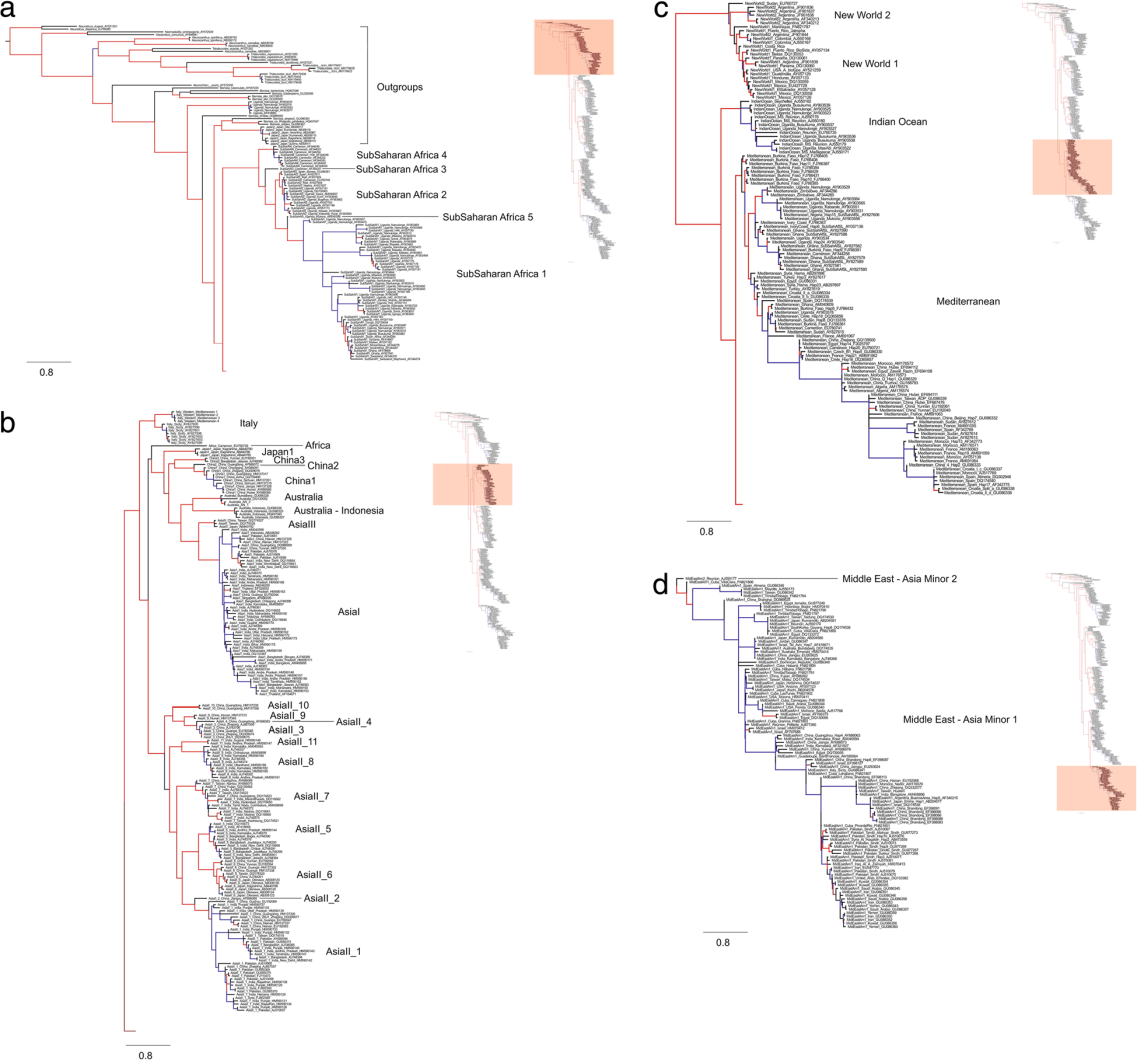
**MrBayes phylogeny for the Aleyrodidae generated using 30 million generations, trees sampled every 1000 generations and 25% discarded as burnin.** The tree has been split into four panels **(a-d)**. The red shading in the summary tree (top right of each panel) indicates where the section fits in the overall tree. The phylogeny containing all samples is shown on the right and the section highlighted in salmon is magnified on the left. Lines are colored based on posterior probability (PP) values generated using MrBayes. Red lines indicate greater than 0.70 PP, black lines represent 0.70 – 0.50 PP, blue lines indicated less than 0.50 PP.

Figure 2 is the global phylogeny for the *B. tabaci* species complex. Starting at the base of the tree, the two species of *Aleurodicus* (subfamily Aleurodicinae) are basal to the Aleyrodinae (Figure 2). *Neomaskellia* and *Vasdavidius* are the basal members of the subfamily Aleyrodineae leading to a dichotomy of *Trialeurodes* (4 species) and *Aleurocanthus* (2 species) comprising a monophyletic group basal to *Aleurochiton aceris* and all other *Bemisia* species (Figure 2). *Bemisia* forms a monophyletic group although not all individuals, morphologically identified as *B. tabaci*, fall within the largely monophyletic grouping that makes up the species complex. Several accessions from Uganda sit within the grouping of a number of non-*tabaci* species, namely *Bemisia tuberculata*, *B. berbericola, B. subdecipiens, B. afer, B. emilae* and *B. atriplex*. In addition, several accessions from Japan AB308117, AB308116, AB240967, AB3080115, AB308118-9, AB308111, identified as *B. tabaci*[48], but these sequences lie outside the ingroup and further investigation is necessary to determine if they are *B. tabaci*. These accessions were referred to as JpL, a new species within the *B. tabaci* complex, but this is unlikely to be the case and represents a failure of the authors to consider the morphology of these individuals and to ensure that they covered the full diversity of the complex. Within the *B. tabaci* ingroup, the sub-Saharan African clade is basal to a large clade, which is split into two sub-clades. The relationships of the putative species in the *B. tabaci* complex are unchanged from the phylogeny generated by Boykin [7], a formal revision of the species complex is pending so the naming used herein is consistent with that being used in the current literature.

### Divergence estimates

**Figure 3:**
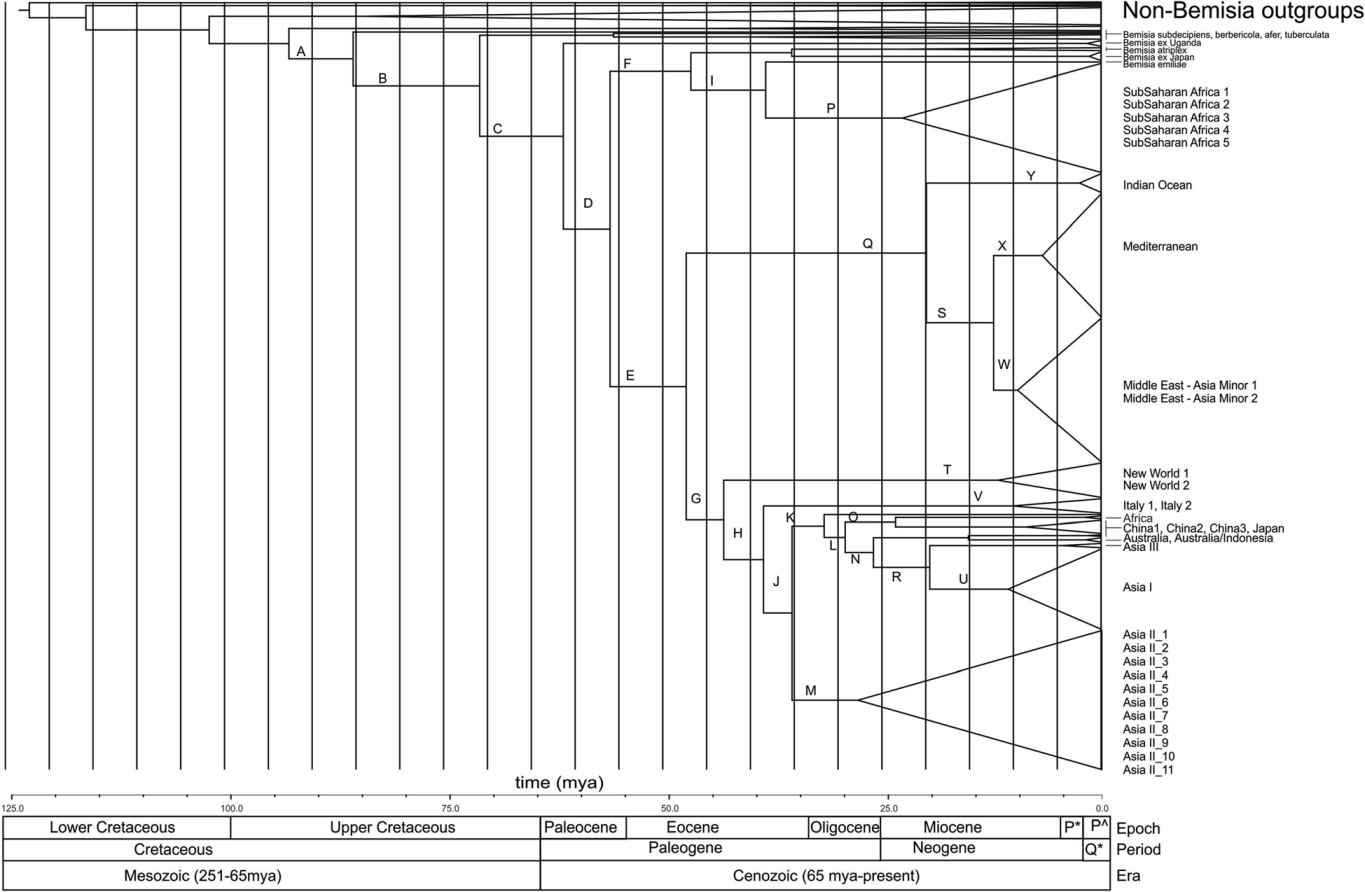
**Summary BEAST tree showing the relationships among the major clades of the *B. tabaci *species complex.** Letters correspond to clades listed in Table 1. Age estimates are shown above the branches. P* = Pliocene, P^=Pleistocene, and Q* = Quaternary.

Table 1 and Figure 3 show the divergence date estimates with confidence intervals for the members of the Aleyrodidae. According to our molecular clock analyses of mtCOI data, the genus *Bemisia* separated from the other members of the subfamily around 86 mya. The topology of the MrBayes tree (Figure 2) and the BEAST tree differ (Figure 3) in the placement of the sub-Saharan Africa species therefore the estimate for the *B. tabaci* species complex split is sometime between 50 and 70 mya (nodes C and D in Figure 3) which encompasses this discrepancy. The majority of the *B. tabaci* species complex diversified between 60–30 mya (Figure 3). The Asian species in the *B. tabaci* species complex went through substantial diversification ~30 mya. The highly invasive species MED and MEAM1 diverged from the Indian Ocean species around 12 mya.

**Table 1:** **Divergence dates of the major clades of the *B. tabaci *species complex estimated using BEAST**[44]**, see Figure**3**for alphabetically labeled clades**

**Clade**	**Taxon**	**Estimated age**	**95% HPD**
A	*Bemisia*	86	70-102
B	Various *Bemisia* species	72	62-89
C	Various *Bemisia* species	62	50-77
D	*B. tabaci* complex	57	45-66
E	All major clades split from the SSA species	48	34-60
F	Sub-Saharan Africa/*Bemisia* sp.	48	30-64
G	New World species and Asian species diverge	44	33-55
H	Asian species and Italy diverge	39	23-53
I	*B. emilae* and sub-Saharan Africa	39	20-48
J	Asian species diversification	36	25-50
K	Asian species diversification	32	22-48
L	Asian species diversification	30	20-44
M	AsiaII diverges	28	18-38
N	Asia1/Australia species diversification	27	17-40
O	China1, 2, 3 and Japan diverge	24	14-45
P	Sub-Saharan Africa	23	12-39
Q	MED/MEAM1/Indian Ocean	21	10-35
R	Asia1/AsiaII/Australia	20	9-35
S	MED/MEAM1	13	8-25
T	New World	13	5-28
U	Asia1	11	4-30
V	Italy	11	6-27
W	MED	10	2-29
X	MEAM1	7	0.5-15
Y	Indian Ocean	3	0.5-8

The highest probability density (HPD) is the credible set that contains 95% of the sampled values.

## Discussion

The divergence dates of the species lineages within the *B. tabaci* complex all pre-date the evolution of *H. sapiens* and agriculture and so human activity is not responsible for the diversity being observed within the complex. The multiple independent runs generated the same age estimates and confidence intervals around each divergence date are well beyond those for the evolution of *H. sapiens*. The approach used is well supported by a number of recent studies [49-51] and adheres to current best practice [33].

In contrast, there are examples where human activity appears to be driving speciation via host race formation [52-55]. In these cases, selection pressures imposed by each plant species is driving diversification despite the overlapping distribution of host plant species. The evolution of specific host races is thought to represent the incipient stage of sympatric speciation which is perhaps best exemplified through the apple maggot fly, *Rhagoletis pomonella* Walsh (Diptera: Tephritidae) [56-58]. In the case of *B*. *tabaci*, there is considerable discussion relating to the apparent variation across the complex in regards to capacity to utilize different host plant species. However, our knowledge of host range across the *B. tabaci* complex is decidedly patchy with much of our knowledge being assumed on the basis of scant comparisons against the highly invasive MED and MEAM1 both of which have a very broad host range. As such, the hypothesis posed by [59], that variation in host plant utilization was driving diversity within *B. tabaci*, has yet to be adequately tested.

The genus *Bemisia* diverged from other members of the subfamily during the Upper Cretaceous around 86 mya. Prior to this, was a period of considerable angiosperm diversification which occurred during the lower Cretaceous, a relatively warm interval in Earth history with elevated levels of atmospheric carbon dioxide [60]. This period (156 – 101 mya) saw the evolution of major lineages within the angiosperms (Mesangiospermae, Gunneridae, Rosideae, and Asterideae) [51]. Given that most members of the Aleyrodidae utilize angiosperm hosts [61], it is likely that the diversification of the angiosperms opened the way for the diversification within the Aleyrodidae. Our divergence estimates support the hypotheses proposed by Campbell [32]. Drawing upon fossil evidence [62], Campbell [32] argued that the two major subfamilies with the Aleyrodidae, the Aleyrodinae and Aleurodicinae, diversified after the lower Cretaceous, but before the Eocene.

**Figure 4:**
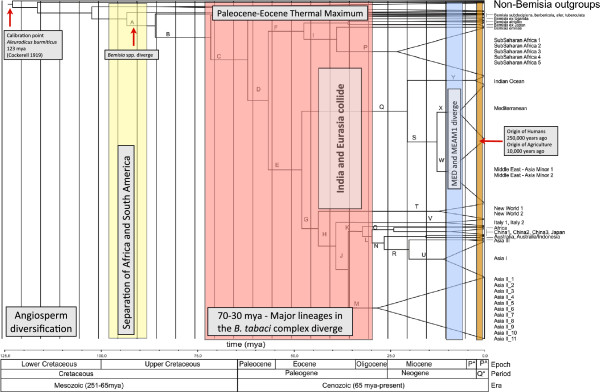
**The divergence estimates (Figure**3**) do not support the view that human activity has been a major driver of diversification.** Rather, the analysis suggests that the complex arose approximately around 80 mya and the highly invasive MED and MEAM1 split from the rest of the species complex around 12 mya well before the evolution of Humans. Major events the correlate to the diversification dates for the *B. tabaci* species comples include: the divergence of Angiosperms, Gondwana breaking apart (South America from Africa approximately 90 mya and India colliding with Eurasia approximately 50 - 30 mya), and Paleocene-Eocene Thermal Maximum.

The majority of the diversification within the *B. tabaci* species complex occurred between 60–30 mya, which corresponds to the Paleocene and Eocene Epochs within the Paleogene Period and the Cenozoic Era (Figure 4). This period corresponds with the Paleocene-Eocene Thermal Maximum (PETM), also called Initial Eocene Thermal Maximum (IETM), which occurred around 55 mya. This period of global high temperatures is thought to have resulted from a period of considerable methane gas release as a result of volcanic activity [63,64]. It is hypothesized that this increase in temperature and atmospheric carbon dioxide was also associated with a marked increase in insect herbivory. The evidence for this latter point comes from leaf fossils which show a marked increase in damage consistent with insect feeding [65]. This overall association between elevated temperatures, carbon dioxide and insect herbivory has also been made by Drohojowka and Szwedo [29] in their description of four new whitefly fossils (*Oisedicus maginus*, *Clodionus fizoli, Lukotekia menae* and *Isaraselis cladiva)* from the Eocene (55–53 mya), a period that also correlates with a marked increase in the number and diversity of Aleyrodidae fossils.

Biogeographically, there are two interesting correlations between divergence dates for the *B. tabaci* species complex and major biogeographic events. Firstly, the divergence date for the branch leading to the entire *Bemisia* genus (node A in Figure 3, approximately 90 mya) coincides with the splitting of the Africa and South American continents [66]. Present geographic distribution of the two major Aleyrodidae subfamilies, Aleurodicinae and Aleyrodinae, coincides with the separation of Gondwana and points to this as the paleoevolutionary event that may have lead to the divergence of these major whitefly subfamilies [32]. The present-day distribution of Aleyrodidae lineages shows that the Aleurodicinae are primarily distributed across the Neotropical and Australasian regions, while the Aleyrodinae have a worldwide distribution [67]. This distributional pattern as well as the Aleyrodidae fossil record [27-29] and molecular data [7,31] supports the Palaeotropical origin of whiteflies [32,61,67]. Secondly, the divergence dates for the Asian species in the *B. tabaci* species complex (approximately 40–30 mya) correlates with the collision between the India and Eurasia plates which occurred somewhere between 50–35 mya. The association between the diversification within the Asian members of the *B. tabaci* complex and the breakup of Gondwana is supported by the presence of an individual haplotype from Cameroon, African (EU760739). This haplotype, referred to as Africa (Figures 2 and 3) is placed at the base of one of the two major Asian lineages at approximately 35 mya.

The diversification within the *B. tabaci* species complex occurred between 60–30 mya, dates which correspond to the Cenozoic Era. This Era is also associated with widespread diversification across plants, insects, fishes and many other organisms [68] and so it would appear that the processes driving the diversification of *B. tabaci* species complex were not unique, but rather were part of an overall pattern of species expansion and diversification.

The origin of the *B. tabaci* species complex is the sub-Saharan region of Africa [7,8,10,32]; the continent continues to suffer from the severe impact that members of this species complex have on crops central to food security within the region. Many countries within sub-Saharan Africa are severely impacted by diseases such as cassava mosaic disease (CMD) and the cassava brown streak disease (CBSD) which are transmitted by members of the *B. tabaci* complex to cassava, a key food security crop [69]. In Uganda alone, an estimated 200 million people are affected [70] by production losses due to these viruses. The species within the *B. tabaci* complex responsible for transmitting these viruses all belong to the sub-Saharan African clade (Figure 3). It has been suggested that the interaction between *B. tabaci*, cassava and viruses infecting cassava has driven the diversification observed within the complex in sub-Saharan Africa [70-72]. Our analysis supports the argument that diversification of *B. tabaci* within Africa occurred well before cassava was introduced there somewhere between the 15th and 17th centuries [73,74]. The first suspected presence of CMD and CBSD in the 1930s [71]. It is interesting to note that the most recent published research on cassava infecting viruses in Africa suggests a far less tightly coupled relationship between the different vectors within the complex and the recent CBSD outbreak [69] suggesting a lack of co-evolution between the vectors and viruses.

## Conclusion

Human activity does influence certain traits expressed by different members of the *B. tabaci* species complex, the most apparent of these is the evolution of insecticide resistance [75-78]. However, the extension of this type of observation to the argument that human activity has driven diversification within the *B. tabaci* complex, cannot be sustained. Diversification within the complex coincided with a period of diversification that occurred broadly across the plant and animal kingdoms and is most likely associated with major climatic and tectonic events. As more fossil and genetic data become available, we will be able to further refine the complex evolutionary history of the *B. tabaci* complex and through this, gain further insights into the evolution of traits that make members of this complex amongst some of the most damaging to agricultural productivity. A sound understanding of the evolution *of B. tabaci* is likely to provide a good foundation upon which to develop long-term sustainable management.

### Data availability

Our alignment is available at the CSIRO data portal: http://dx.doi.org/10.4225/08/50EB54B6F1042. Treebase http://purl.org/phylo/treebase/phylows/study/TB2:S14655. Genbank accession numbers are listed on the tips of Figure 2.

## Competing interests

The authors declare we have no competing interests.

## Authors’ contributions

LMB, PJDB, and GE designed the study. GE conducted morphological comparative analyses. LMB and CB carried out the BEAST analysis. CB and IS advised on angiosperm evolution and molecular rates of evolution. LMB, PJDB, GE, CB and IS drafted the manuscript. All authors read and approved the final manuscript.
